# Pupil responses to implied motion in figurative and abstract paintings

**DOI:** 10.1371/journal.pone.0258490

**Published:** 2021-10-11

**Authors:** Serena Castellotti, Lisa Scipioni, Stefano Mastandrea, Maria Michela Del Viva

**Affiliations:** 1 Department of Neurofarba, University of Florence, Florence, Italy; 2 Department of Education, Roma Tre University, Florence, Italy; UNSAM Escuela de Ciencia Y Tecnologia: Universidad Nacional de San Martin Escuela de Ciencia Y Tecnologia, ARGENTINA

## Abstract

Motion can be perceived in static images, such as photos and figurative paintings, representing realistic subjects in motion, with or without directional information (e.g., motion blur or speed lines). Motion impression can be achieved even in non-realistic static images such as motion illusions and abstract paintings. It has been shown that visual motion processing affects the diameter of the pupil, responding differently to real, illusory, and implied motion in photographs (IM). It has been suggested that these different effects might be due to top-down modulations from different cortical areas underlying their processing. It is worthwhile to investigate pupillary response to figurative paintings, since they require an even higher level of interpretation than photos representing the same kind of subjects, given the complexity of cognitive processes involved in the aesthetic experience. Also, pupil responses to abstract paintings allows to study the effect of IM perception in representations devoid of real-life motion cues. We measured pupil responses to IM in figurative and abstract artworks depicting static and dynamic scenes, as rated by a large group of individuals not participating in the following experiment. Since the pupillary response is modulated by the subjective image interpretation, a motion rating test has been used to correct individual pupil data according to whether participants actually perceived the presence of motion in the paintings. Pupil responses to movies showing figurative and abstract subjects, and to motion illusions were also measured, to compare real and illusory motion with painted IM. Movies, both figurative and abstract, elicit the largest pupillary dilation of all static stimuli, whereas motion illusions cause the smallest pupil size, as previously shown. Interestingly, pupil responses to IM depend on the paintings’ style. Figurative paintings depicting moving subjects cause more dilation than those representing static figures, and pupil size increases with the strength of IM, as already found with realistic photos. The opposite effect is obtained with abstract artworks. Abstract paintings depicting motion produce less dilation than those depicting stillness. In any case, these results reflect the individual subjective perception of dynamism, as the very same paintings can induce opposite responses in observer which interpreted it as static or dynamic. Overall, our data show that pupil size depends on high-level interpretation of motion in paintings, even when they do not represent real-world scenes. Our findings further suggest that the pupil is modulated by multiple top-down cortical mechanisms, involving the processing of motion, attention, memory, imagination, and other cognitive functions necessary for enjoying a complete aesthetic experience.

## Introduction

The pupillary light reflex (PLR) controls the pupil diameter by constricting or dilating the pupil in response to light changes, in order to regulate retinal illumination and optimize vision [1–4]. It has been shown that the pupil also responds to extra-retinal factors, revealing that the PLR is not an automatic low-level mechanism that merely regulates the intensity of the light entering the eye [5–7].

Previous evidence showed that pupillary dilation can be induced not only by light decrements but also by cognitive factors associated with the activation of the sympathetic system [7,8]. For example, pupillary responses have been successfully used as index of emotional processing and arousal [9–12], mental activity and cognitive load [13–15], memory creation and retrieval [16–21].

Moreover, several studies demonstrated that pupil size is not solely determined by physiological factors but also by high-level visual processes not linked to increased arousal [22]. For instance, pupil constriction has been found not only in response to light increments but also in response to brightness and contrast changes in binocular rivalry conditions [23–26], onset of low-level features changes [27,28], lightness or color visual illusions [29–31], covert attention to light [32–34], imagery of brightness [29], and interpretation of luminous objects depicted in photos or paintings [35–37].

Visual motion analysis, consisting in the processing of luminance and contrast changes over time and space, has been also suggested to exert an influence on pupil diameter, as well as the perception of dynamism from static images (illusory motion and implied motion—IM) [28,38,39]. Particularly, it has been shown that movies with real moving subjects, optical illusions of motion, and realistic photos representing dynamic scenes (implied motion) elicit different pupillary responses, maybe related to different top-down modulation [38].

The movement investigated through the medium of photography is given by images that capture reality. However, the perception of movement can also be communicated by a class of stimuli that belong to a special category called *art*, such as paintings and sculptures, which express different kinds of contents according to the point of view of the artist, his style, and his vision of the world [40]. In different forms of visual arts the viewers can have the impression that what they see is a depiction of real locomotion, although artworks can be either faithful and accurate illustrations or even highly distorted representations of reality [40,41].

In figurative artworks the perception of motion can be attributed to several factors: dynamic balance or instability (e.g., a picture breaking bilateral symmetry), multiple images (e.g., several pictures of the same objects shifting horizontally or overlapping one another), affine shear (e.g., objects inclined and in a diagonal position), blur (e.g., a fuzzy picture); vector-like lines (e.g., streaks superimposed on an image indicating motion direction) [42], extension (e.g., animals’ legs extension during gallop) [43]. Some of these techniques, used to enhance motion impression, are unrealistic: indeed, in real-life conditions, given the spatiotemporal properties of our motion mechanisms, we do not see blur, motion strikes, and lines when something moves in the scene [44,45].

Besides, in artworks, we are able to grasp the presence of movement even if they that do not represent the real world, such as abstract paintings. Abstract art is characterized by the presence of structural features such as lines, shapes, colors and materials, and compositional characteristics such as balance, symmetry, orientation, etc., where the representation of everyday objects is absent. Thus, in the abstract artworks, the perceived dynamism does not derive from the intrinsic realistic characteristics of the depicted object (a running man, galloping horse, waterfall, etc.), but from the use of different techniques playing with lines, shapes, and directions on the bi-dimensional surface [46]. For example, patterns with acute angles presented as diagonals or wedge shapes in oblique position [47], shapes with curvy and wavy lines [48], multiple and successive stroboscopic images superimposed on a flat surface [49], might convey the sense of movement.

Whatever factor is used to describe the movement, the observer grasps the dynamism communicated by the artworks and usually gives a positive aesthetic appreciation [46,47]. However, generally, non-expert observers show a preference for figurative art compared to abstract one [50–53]. In addition, familiarity with an art style plays an important role in its appreciation [54–56]. It has been also shown that familiarity results in a faster processing and higher preference for the familiar stimuli compared to the novel ones [57–59]. Finally, expertise in art facilitates the so-called *aesthetic fluency* [60]; a process that could lead people to better grasp the aspects related to the movement in an artwork and to its aesthetic appreciation. Very few studies have tried to relate the pupillary responses with aesthetic pleasantness or familiarity, generally finding pupillary dilation for pleasant and familiar stimuli [21,61–63].

Here, for the first time, we study if pupil diameter is influenced by the perception of motion in paintings, which require a greater interpretative effort and a greater involvement of multiple cognitive dimensions (attention, imagination, familiarity, memory, pleasantness, etc.) compared to photographs [40,64–67]. Furthermore, unlike in photography, abstract art offers the advantage to study the influence of implied motion on the pupil using geometrical stimuli devoid of representational subjects or real-life motion cues. Given the diversity and complexity of our stimuli we expect to find different pupil responses.

We measured pupillary responses to artistic representations of implied motion by presenting figurative and abstract paintings belonging to different styles and different historical periods. To assess the strength of the responses to IM paintings, these were compared to those induced by real motion of abstract and figurative subjects. Figurative stimuli included either humans and objects/animals, that have shown to induce different pupil responses [38]. We also measured pupillary responses to illusory motion stimuli, that are images devoid of representational subjects and have been shown to induce a different pupil modulation compared to photographs with IM and real movies [38]. The comparison of the response to illusory stimuli with those to abstract paintings giving the impression of motion will provide insights about the mechanisms subtending perception of motion devoid of figurative content. Since the subjective interpretation of the stimuli modulates the pupil size of the observers [37,38,68], pupillary responses to each painting were corrected based on participants’ perception of dynamism or staticity in the scene by means of a motion rating test. Given the complexity of our stimuli, participants’ judgment about aesthetic pleasantness and familiarity for each painting was also collected, in order to control possible influences of these factors on the pupillary responses of the observers.

## Materials and methods

### Participants

Twenty-four subjects, all non-expert in art, participated in the experiment (mean age = 25 years, SD = 4). Before starting the pupillary data collection, information about personal data and art expertise (rated with a 5-point Likert scale) of participants were collected. All participants had normal vision or corrected by contact lenses and were naïve to the purpose of the experiment. None of our participants was a painter, and, on average, they reported to have *little* artistic experience (mean score = 2.3, SD = 0.9) and to have visited museums or art exhibitions only 1 or 2 times in the last year. Participants gave written informed consent prior the participation. The experimental procedures were approved by the local ethics committee (Comitato Etico Pediatrico Regionale—Azienda Ospedaliero-Universitaria Meyer—Firenze FI) and were compliant with the Declaration of Helsinki.

### Apparatus and set up

All measurements were recorded in the dark and observers’ heads were supported by a chin- and forehead-rest. Stimuli were presented on a 51 x 29 cm ASUS monitor at a fixed viewing distance of 57 cm. The refresh rate of the screen was 60 Hz, and the resolution was set to 1920 x 1080 pixels. Left and right pupils’ diameter was tracked with a CRS LiveTrack FM system (Cambridge Research Systems) at 60 Hz. During the whole pupil recording, the experimenter controlled the corrected position of the observers’ eyes by means of a camera (QuickTime software). Stimuli were generated on a Mac computer (iMac Retina 5K, 27-inch, mid 2015 3.3 GHz Intel Core i5 processor, MacOs Sierra software 10.12.6) and presented using using the Psychophysics Toolbox extensions [69–71] for Matlab (R2016b version). Data were analyzed by JASP software (Version 0.8.6).

### Stimuli

A wide variety of stimuli were used in this study: 80 paintings, realized in different historical periods and different styles, 20 movies and 10 optical illusions.

The 80 paintings in the final set have been selected from an initial pool of 200 images, which were categorized by 50 naïve judges (different from the participants to the experiment) as static or dynamic representations. Images that did not reach at least 70% agreement on the judgment as static or dynamic were excluded from the pool of possible experimental stimuli. Paintings that reached the highest percentage of agreement among the judges were included in the chosen set of stimuli. For a complete list of all paintings used see S1 List.

**Fig 1:**
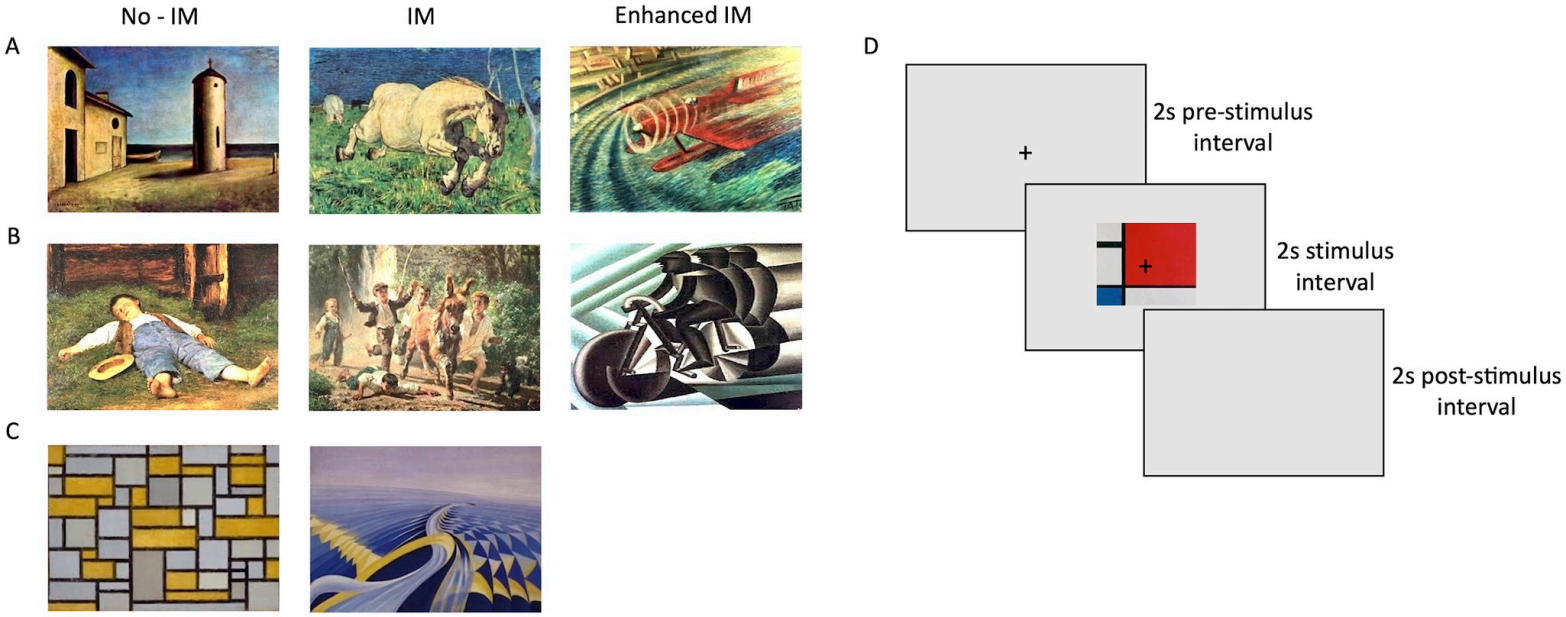
Paintings’ categories and procedure. **(A)** Examples of figurative paintings representing objects or animals. From left to right: *Dopo il tramonto* (Carrà, 1927); *Cavallo al galoppo* (Segantini, 1858); *Idrovelocità*: *folgore rossa* (Sansoni, 1935); **(B)** Examples of figurative paintings representing humans. From left to right: *Sleeping boy in the bay* (Anker, 1891); *Monelli di strada* (Palizzi, 1872); *Ciclista* (Depero, 1922); **(C)** Examples of abstract paintings. From left to right: *Composizione con grigio e ocra* (Mondrian, 1918); *Velocità di motoscafo* (Cappa Marinetti, 1922). Paintings shown are in the public domain. Copyright laws prohibit reproduction of all paintings used as stimuli, but they can be viewed on the web using the links reported in S1 List. **(D)** Schematic representation of a trial.

Paintings and movies representing recognizable images of the world around us were considered as “figurative stimuli”. Figurative paintings were assigned to three different nominal categories according to the depicted motion intensity: the “No-Implied Motion” category (No-IM) consists of 20 paintings of static scenes (such as a man sitting or a vase of flowers on a table); the “Implied Motion” category (IM) consists of 20 depictions of dynamic subjects (such as a woman dancing or the waves of a stormy sea); the “*Enhanced* Implied Motion” category (E-IM) consists of 20 paintings depicting blurred subjects or backgrounds (such as a blurry racing car or a runner with motion streaks in the direction of the movement) (for examples see Fig 1A and 1B). In each motion category, 10 paintings depict human figures and the other ten animals/objects. Figurative real motion stimuli were 10 short clips (2 sec) of movies, 5 containing moving people (e.g., cyclists in a race) and 5 moving objects/animals (e.g., a running horses).

Paintings and movies depicting non-representational images (shapes, forms, marks, etc.), with no derivation from real figures or objects, were considered as “abstract stimuli”. Abstract paintings were assigned to two different nominal categories according to the depicted motion intensity: the “No-Implied Motion” category (No-IM) consists of 10 representations conveying a sense of stillness (i.e., by symmetric patterns arrangement); the “Implied Motion” category (IM) consists of 10 representations conveying a sense of dynamism (i.e., by asymmetric and curved shapes) (for examples see Fig 1C). Abstract real motion stimuli were 10 short clips (2 sec) of movies, containing squares of different colors moving right-to-left of left-to-right on a grey background, purposedly created by the authors.

Optical illusions, which make up the “Illusory Motion” category, are 10 color-full images retrieved from internet, whose features were specifically manipulated by the illusions’ creators to induce the impression of motion. The set of illusions was the same as that used in a previous experiment [38]. Motion illusions can be, in some way, suitable stimuli to be compared with abstract paintings, being non-objective images without any realistic recognizable representation but still conveying a strong impression of motion trough several structural characteristics.

All experimental stimuli were resized, without modifying proportions, to have either a width or a height of 340 pixels (the other side ranged from 186 to 420 pixels). The original mean luminance of each stimulus was changed and adjusted to be the same for all of them and the same of the stimuli used in our previous work on photographs (25 cd/m^2^) [38]. This adjustment allows us to make comparison between pupillary responses to different types of motion and between motion in photos and paintings.

### Procedure

The whole session consisted in the presentation of 110 stimuli, of which 90 images, divided into five blocks, and 20 movies (presented in a single block). At the beginning of the block, the eye tracker was calibrated with a standard 9-point calibration routine. The stimuli presentation order was generated using a random sequence and was the same for all participants. Each trial started with the presentation of a black fixation cross (5 x 5 mm) for two seconds, centrally located in a white screen (124 cd/m^2^). Then, one of the stimuli appeared for two seconds in the center of the screen, with the fixation cross still visible. The luminance of the background was the same of the fixation interval. Observers were asked to process the content of the presented stimulus without performing any other task, and they were instructed to refrain from blinking. To avoid any effect of eye movements on pupil size, participants were asked to fixate on the screen centre for the entire length of the experiment. Fixation was also monitored by the experimenter (see Apparatus and set-up section). Every stimulus presentation was followed by a blank screen (124 cd/m^2^) of two seconds, during which pupil recording was interrupted and participants could blink and rest their eyes before the next trial (Fig 1D). The post-stimulus interval was sufficiently long to prevent the build-up of after-images. Every two blocks, observers were allowed to take a break of about 5 minutes.

Once the experiment is over all the images were presented again in sequence to the participants, without pupil recording. For each image the observers had to evaluate on a three-point scale; 1) how much motion they perceived in the scene (0 = no motion, 1 = low motion, 2 = high motion); 2) how much they liked it (0 = not at all, 1 = a bit, 2 = a lot); 3) if they have already seen it prior to the experiment (0 = No, 1 = Maybe, 2 = Yes). In order to simplify the data analysis, the participants’ answers were scored and classified in two possibilities only. For Question 1 the answer was considered as “perception of staticity” if the participants reported to not perceive motion in the image at all (score 0), and as “perception of dynamism” if they reported to perceive low or high motion (score 1 or 2). Question 1 was used as *motion rating test* to correct observers’ pupil data (see Data processing section). For Question 2 the answer was considered as “unpleasant” if the participants reported not to like the picture at all (score 0), and as “pleasant” if they reported to like it, both a bit or a lot (score 1 or 2). For Question 3 the answer was considered as “unfamiliar” if the participants reported to have never seen the stimulus (score 0), and as “familiar” if they report to have already seen it before the experiment (score 2), whereas the answer was excluded if the participants reported to be doubtful about their familiarity with the stimulus (score 1). The total time required for the whole experiments was approximately 1 hour.

### Data processing

The eye-tracker measured the width and the height of both pupils in pixels. Right and left pupil diameters were then averaged, and the resulting value was converted from pixels to millimeters, based on the instrument’s recording of a 4 mm artificial pupil, positioned at the location of the observer’s left eye [36–38].

Pupil responses elicited by different categories of stimuli were analyzed following a widely-used method for this type of experiments [35–37]. For each observer, a baseline pupil diameter was calculated by averaging pupil diameter recorded over the last 500 ms of the fixation slide in each trial. This baseline was subtracted from each recording of that observer over the whole 4 sec period [37,72]. Baseline-corrected pupil size as a function of time of each observer was calculated by averaging the pupil responses for the ten stimuli of each category. Final traces for each category were then obtained by averaging all observers’ traces.

The average pupil size of each observer for each image/video was calculated by averaging the baseline-corrected pupil values during the stimulus presentation (120 data points). Then, for each observer, these values were averaged over all ten stimuli of the same category. However, data have been corrected based on the participants’ answers to the motion rating test (Question 1 of the final questionnaire, see Procedure section) and pupillary responses to stimuli that participants did not categorize in agreement with our nominal classification were considered as "miscategorizations" and were excluded from the analysis. That is, if participants perceived motion (score 1 or 2 on Question 1) in some paintings nominally categorized in the “No-Implied motion” category, or if they did not perceive motion (score 0) in paintings nominally categorized in the “Implied motion” or “*Enhanced* IM” category, their answers were considered as “miscategorizations”. Finally, the mean pupil responses for each category were obtained by averaging the responses over all observers. Differences between the means of categories were assessed with ANOVAs, and pairwise comparisons were done with post-hoc t-tests (Bonferroni corrected). The size effect of differences between categories was evaluated by Cohen’s d statistics [73,74]. Kruskal-Wallis tests have been used to compare pupillary responses of observers which categorized differently the same stimuli. Significant differences between the percentages of miscategorization, aesthetic pleasantness, and familiarity, across the stimuli categories, were identified through the Chi-squared test.

The eye-tracker also recorded the observer’s eye position along the horizontal and vertical axes. To control fixation, for all categories, we measured the average distance of the eyes, with respect to fixation, of all subjects during stimulus presentation in all trials. ANOVA test was used for comparisons between average distances of categories.

## Results

Given that all types of stimuli have the same average luminance, which is lower than the luminance of the screen during the fixation interval, we would expect the same pupillary dilation for all of them. Instead, either the pupil traces and the average dilation differ significantly across motion categories and art styles.

**Fig 2:**
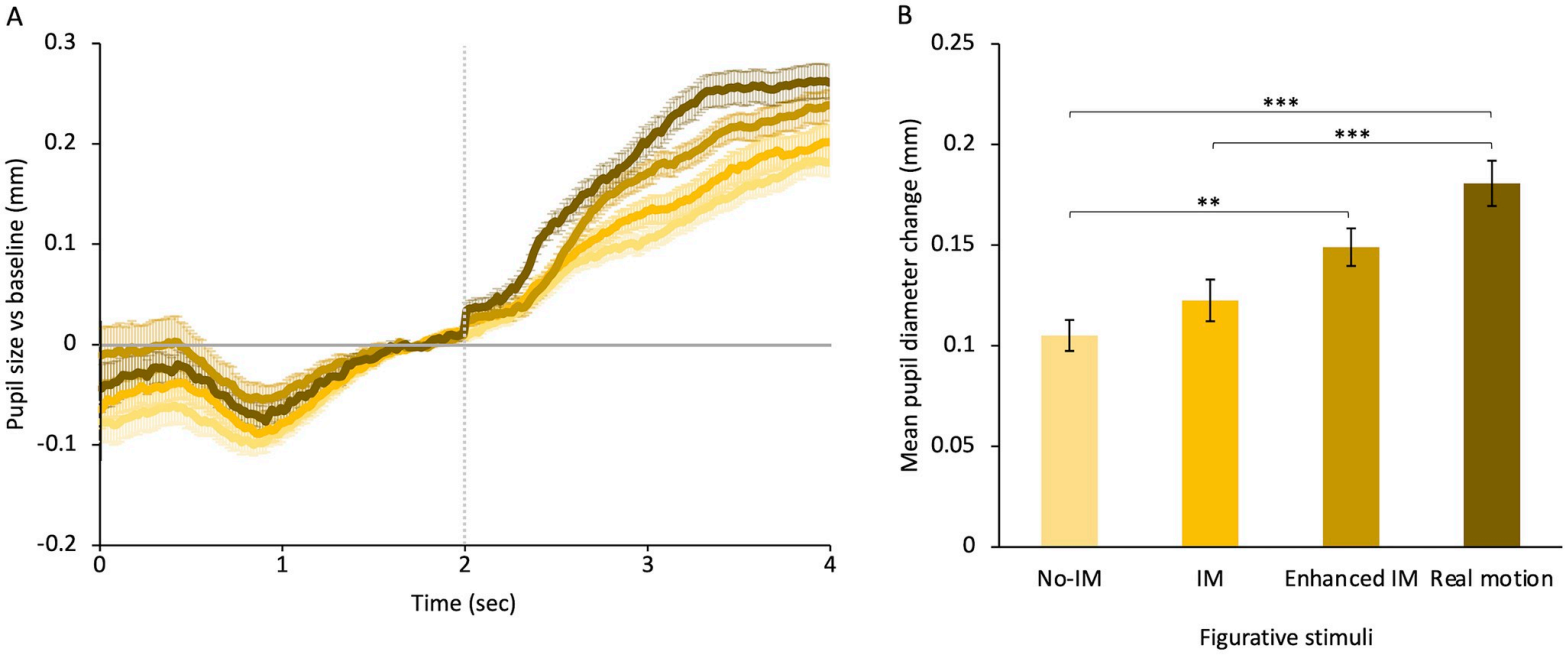
Pupillary responses to motion in figurative stimuli. **(A)** Baseline-corrected pupil size for the different figurative stimuli plotted as a function of time from trial onset. The horizontal line represents the baseline: Data over the zero-line represent pupil dilation whereas data under the line represent pupil constriction. The vertical line indicates the stimulus onset. Error bars are SE. **(B)** Mean pupillary diameter for different figurative categories during stimulus presentation. Categories include both objects/animals’ and humans’ subjects. Error bars are SE. Color saturation of the bars increases with motion strength, in order: Paintings with no implied motion (No-IM), paintings depicting simple implied motion (IM), paintings depicting *enhanced* implied motion (E-IM); movies of moving subjects (Real motion). See examples of paintings for each category in Fig 1A and 1B. Asterisks mark statistically significant pairwise comparisons across figurative motion categories: *** *p* < 0.001, ** *p* < 0.01. All data shown have been corrected based on each observer’s motion rating test.

Fig 2 shows results for figurative stimuli regardless of their content (human vs non-human). Pupil responses over time show that figurative movies induce an increasing pupil dilation for about a second, then the diameter remains almost stable for the rest of stimulus presentation, as found previously [38]. Instead, pupil responses to figurative paintings, regardless of whether they represent motion or not, induce a gradual dilation for the whole time of image presentation (Fig 2A). Mean pupil dilation increases with the amount of motion in the stimulus (Fig 2B). A Two-way ANOVA analysis—type of motion (four levels: No-IM, IM, *Enhanced* IM, and real motion) and type of content (two levels: human or non-human subjects) shows a significant main effect of type of motion (F(3,69) = 13.15, *p* < 0.001, η^2^ = 0.16). Post-hoc comparisons (Bonferroni correction) between motion categories show that real motion in representational movies causes the largest dilation (M = 0.18 ± 0.01), larger than that produced by *enhanced* IM (M = 0.15 ± 0.01, *d* = 0.4, *small* effect), and significantly larger than simple IM (M = 0.12 ± 0.01; *t*(3) = -4.494, *p* < 0.001; *d* = 0.9, *large* effect) and no-IM (M = 0.11 ± 0.01; *t*(3) = -5.50, *p* < 0.001; *d* = 1.1, *large* effect). *Enhanced* IM, in turn, induces larger pupil size than IM (*d* = 0.2, *small* effect) and even significantly larger dilation than no-IM (*t*(3) = 3.66, *p* < 0.01; *d* = 0.5, *medium* effect). Finally, IM induces larger dilation than no-IM although not significant (*d* = 0.2, *small* effect). The difference of dilation between static and all dynamic paintings, obtained by pooling together responses to IM and *Enhanced* IM paintings (M = 0.14 ± 0.01), results to have a *medium* effect size (*d* = 0.6). Pupil responses to stimuli representing humans do not differ from that to stimuli showing objects or animals (F(1,23) = 0.61, *p* > 0.05, η^2^ = 0.001). No interaction between the two factors—type of motion and type of content—is found (F(3,69) = 0.10, *p* > 0.05, η^2^ = 0.001).

**Fig 3:**
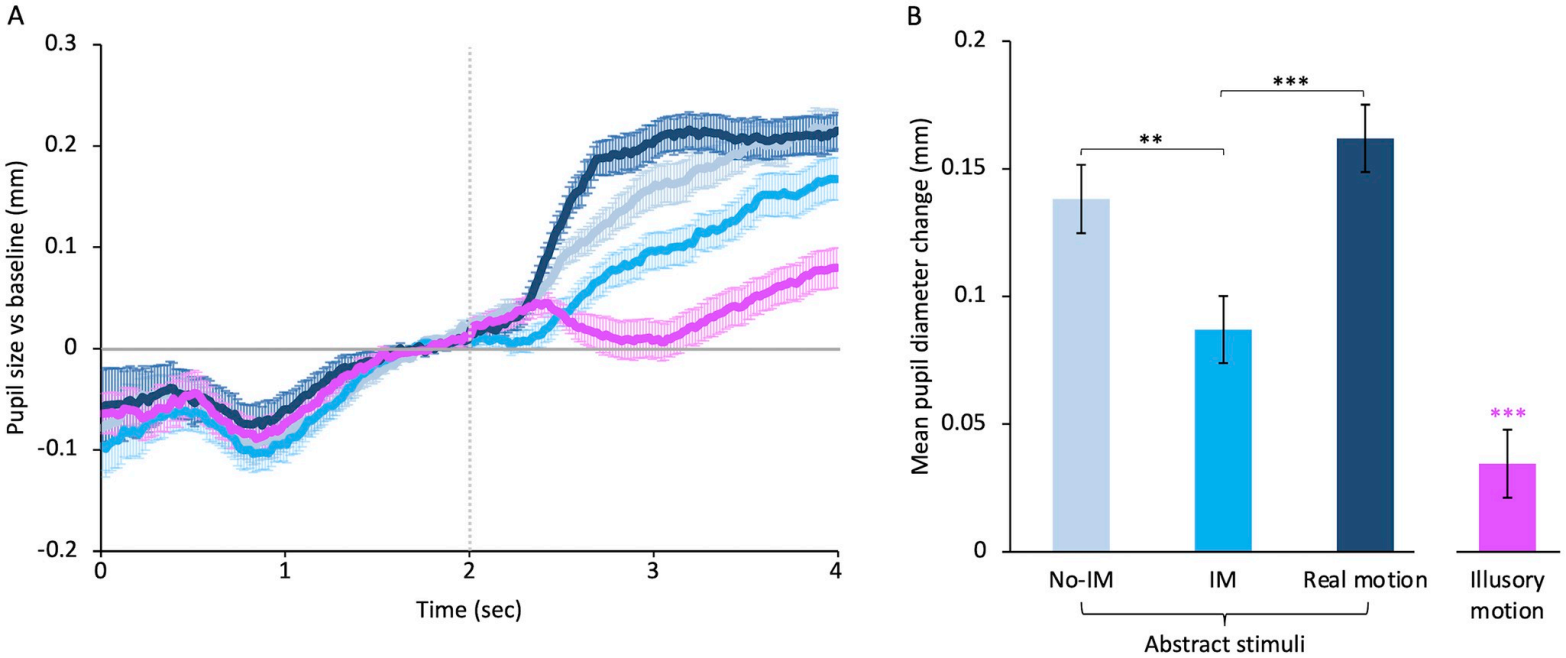
Pupillary responses to motion in abstract stimuli and to illusory motion. **(A)** Baseline-corrected pupil size for the different abstract stimuli and motion illusions plotted as a function of time from trial onset. The horizontal line represents the baseline: Data over the zero-line represent pupil dilation whereas data under the line represent pupil constriction. The vertical line indicates the stimulus onset. Error bars are SE. **(B)** Mean pupillary diameter for different abstract categories and motion illusions during stimulus presentation. Error bars are SE. Color saturation of bars for abstract stimuli increases with motion strength, in order: Paintings with no implied motion (No-IM), paintings depicting simple implied motion (IM); movies of moving objects (Real motion). Purple bar: Illusory motion. Examples of paintings of each abstract category are shown in Fig 1C. Black asterisks mark statistically significant pairwise comparisons across abstract motion categories; pink asterisks denote significant differences with illusory motion: *** *p* < 0.001, ** *p* < 0.01. All data shown have been corrected based on each observer’s motion rating test.

Abstract stimuli of different motion categories also induce different pupillary responses (Fig 3). After presentation of abstract movies pupil diameter rapidly increases with time and then stops increasing, while responses to paintings keep increasing during all stimulus viewing, as for figurative stimuli (compare Fig 3A with Fig 2A). Motion illusions, instead, induce a very different pupil response, as observed previously [38]. After about 300 ms from stimulus onset, pupil size for these stimuli decreases until reaching baseline values and then increases for the rest of stimulus viewing (Fig 3A). Different abstract categories induce different mean pupil diameters as shown by a One-Way ANOVA—with factor type of motion (3 level: No-IM, IM, and real motion)–(F(2,46) = 17.67, *p* < 0.001, η^2^ = 0.20), but the dilation does not increase with the motion strength as for figurative paintings (Fig 3B). Abstract real motion causes the largest dilation of all (M = 0.16 ± 0.1), significantly larger than that produced by abstract IM (M = 0.09 ± 0.1; *t*(2) = -5.38, *p* < 0.001; *d* = 1.1, *large* effect) and slightly larger than that induced by abstract No-IM (M = 0.14 ± 0.1; *d* = 0.4, *small* effect). Pupil diameter in response to abstract IM resulted significantly smaller than that produced by abstract No-IM (*t*(2) = -4.04, *p* < 0.01; *d* = 0.8, *medium* effect). The mean pupil size in response to illusory motion (M = 0.03 ± 0.1) is much smaller than that induced by all the other abstract stimuli (F(3,69) = 34.86, *p* < 0.001, η^2^ = 0.40, all t-tests post-hoc comparisons are significant with *p* < 0.001).

The comparison between stimuli in the two art styles (abstract vs figurative), does not highlight any significant effect of the style (F(1,23) = 1.23, *p* > 0.05, η^2^ = 0.003), but only of motion (F(2,46) = 29.35, *p* < 0.001, η^2^ = 0.17).

There is only one important difference though, while figurative artworks with IM induce an increase of pupil size with respect to their no-IM controls, IM in abstract artworks induce a significant reduction of pupil size with respect to no-IM. With motion illusions this reduction is even stronger.

Although our observers were instructed to keep fixation, motion viewing in the stimuli could trigger eye movements [75,76], which in turn could influence pupil size [77]. For this reason, we measured the average position of subjects’ eyes with respect to the fixation cross, for all the different stimulus categories. The average distance from fixation is minimal (Figurative stimuli—No-IM: 2.14 ± 0.3 mm, IM: 2.44 ± 0.4 mm, E-IM: 1.86 ± 0.4 mm, Real motion: 3.09 ± 0.5 mm; Abstract stimuli—No-IM: 2.42 ± 0.3 mm, IM: 2.61 ± 0.4 mm, Real motion: 2.62 ± 0.4 mm; Illusory motion: 2.55 ± 0.4) and the same for all categories (ANOVA, F(7,161) = 1.36, *p* > 0.05).

**Fig 4:**
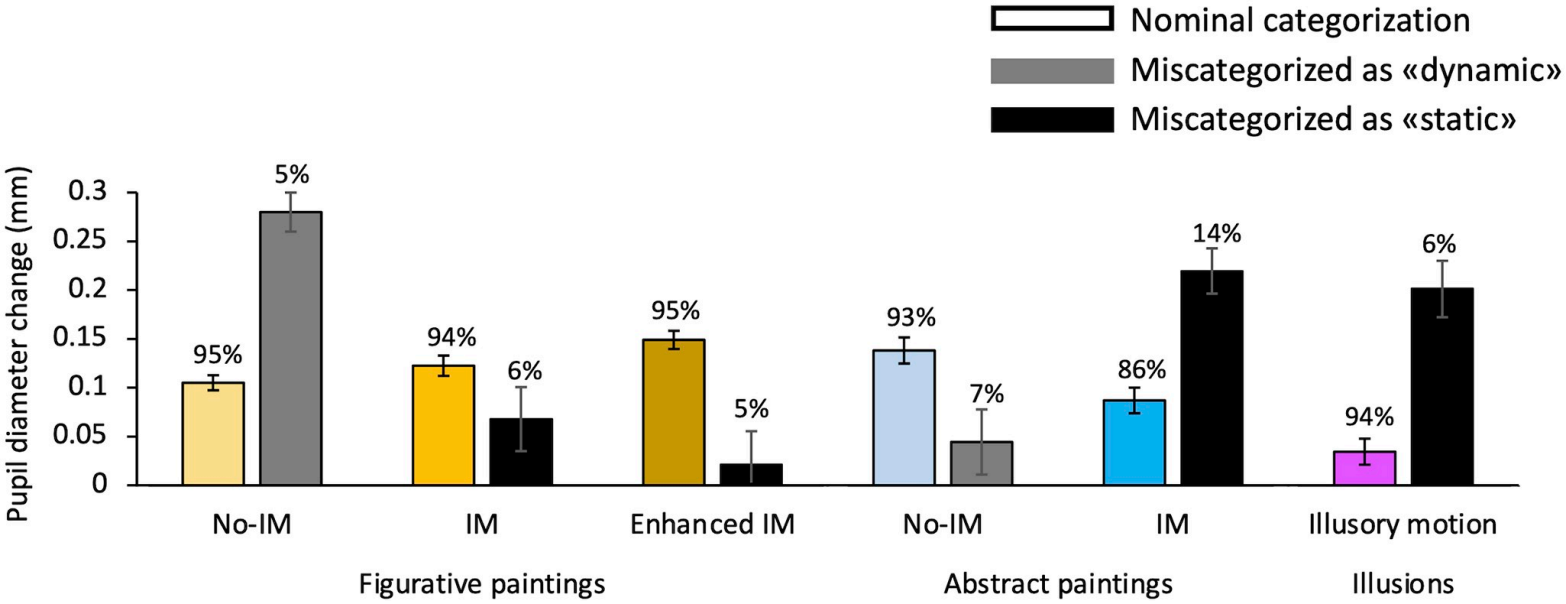
Effects of motion interpretation on pupil size. Mean pupillary diameter for paintings/illusions correctly categorized in accordance with our nominal categorization (colored bars with color-code used in Figs 2 and 3) versus miscategorized stimuli (gray bars: Images rated as dynamic; black bars: Images rated as static). Data of miscategorized paintings/illusions are those excluded from the analysis of pupil responses to each category. Errors bars are SE. Percentages of correct categorization/miscategorization of dynamism for each stimulus category are reported over each bar.

**Fig 5:**
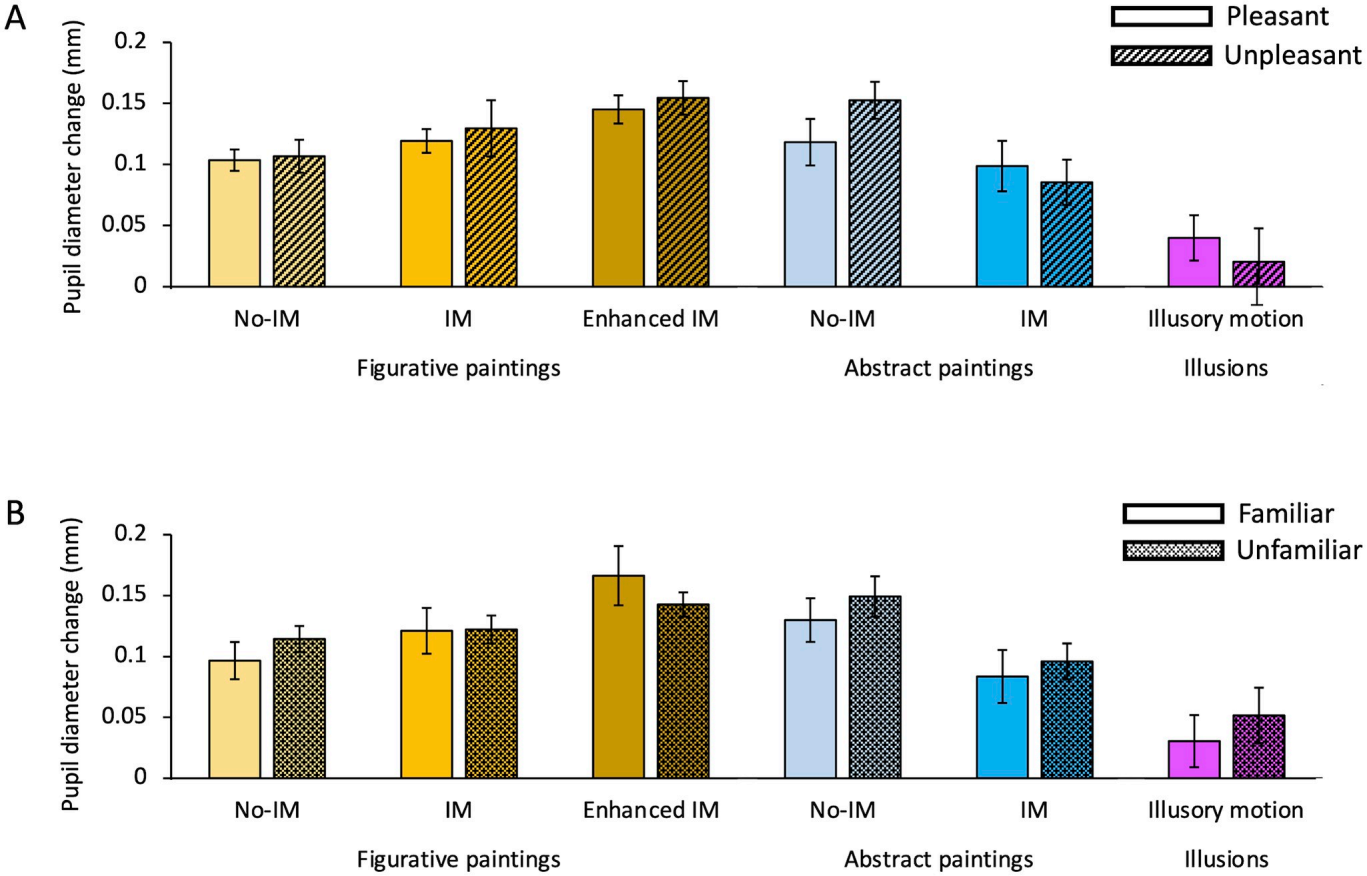
No effect on pupil size of aesthetic pleasantness and familiarity. (A) Mean pupillary diameter for paintings/illusions judged as pleasant by our participants (full-colored bars) versus stimuli considered unpleasant (colored bars with diagonal lines texture). Data reported are the same reported in Figs 2B and 3B (same color-code of bars) but divided based on aesthetic pleasantness. Errors bars are SE. Percentages of pleasantness for each stimulus category are reported over each bar. No significant differences between the mean pupil sizes for pleasant vs unpleasant stimuli. (B) Mean pupillary diameter for paintings/illusions recognize as familiar by our participants (full-colored bars) versus stimuli considered unfamiliar (colored bars with crossed-lines texture). Data reported are the same reported in Figs 2B and 3B but divided based on familiarity (same color-code of bars). Errors bars are SE. Percentages of familiarity for each stimulus category are reported over each bar. No significant differences between the mean pupil sizes for familiar vs unfamiliar stimuli.

Further analyses were conducted to exclude possible effects of variables other than motion on the pupil size. As described in the Procedure section, after the pupillary recording our observers filled out a questionnaire in which they reported how much motion they perceived in each scene, how much they appreciated each picture and whether they already known the stimuli. All our data have been reanalyzed according to individual answers to the questionnaire and are reported in Figs 4 and 5.

In Fig 4, for each category, the mean pupillary response to paintings categorized in accordance with our nominal categorization, already shown in Figs 2 and 3, is shown with that in response to the miscategorized stimuli, that were excluded from the analysis shown before. The percentages of nominal categorizations vs miscategorizations are also shown for each category. The proportion of miscategorizations in each category of figurative paintings is about 5%. That is the large majority of participants correctly interpreted the paintings of the No-IM category as “static” and those of the IM and E-IM categories as “dynamic”. Within figurative paintings, no differences between percentages of miscategorization of human vs non-human representations are found in each motion category (all *X*^*2*^ tests yielded to *p* > 0.05). Similarly, abstract paintings of the No-IM category are miscategorized only in 7% of cases. Instead, a big percentage of miscategorizations occurs for the IM abstract category (14%), which results statistically greater than all the other conditions (all *X*^*2*^ tests yielded to *p* < 0.05). This means that there is less agreement between participants about the content represented in the dynamic abstract paintings, which, therefore, are considered the most ambiguous. Also, there is a small percentage of cases (6%) in which the participants do not perceive the illusion of apparent motion.

Pupil responses of observers that interpreted the same stimulus as static or dynamic are clearly different. Kruskal-Wallis tests have been used to assess the significance of these differences. For figurative paintings, the pupil size of participants that perceived dynamism in paintings nominally representing static subjects is larger than the size of those who correctly categorized them (H(2) = 34.86, *p* < 0.001) as static. Conversely, pupil size of participants that interpreted paintings of IM or E-IM categories as representing static scenes is lower compared to that of those who categorized them as dynamic (H(2) = 5.89, *p* < 0.05; H(2) = 11.29, *p* < 0.001). The very opposite trend is observed in the abstract condition: static paintings interpreted as dynamic produce less dilation with respect to those correctly categorized (H(2) = 6.65, *p* < 0.01), whereas dynamic paintings interpreted as static induce more dilation than those judged in accordance with their nominal categorization (H(2) = 25.78, *p* < 0.001). Also, the absence of motion perception in illusion induces a larger dilation than the perception of illusory motion (H(2) = 16.99, *p* < 0.001). These results clearly show an influence of the subjective interpretation of motion on the pupil, always consistent with the response to motion for the specific style: dilation for figurative motion and constriction for abstract motion.

In Fig 5A mean pupil responses have been replotted, for each category, according to the subjective aesthetic pleasantness. The percentage of aesthetic pleasantness for each category highlights differences between categories and styles. Static and IM figurative paintings are generally considered pleasant and to the same extent (75% and 77% respectively). Figurative paintings with *Enhanced* IM are less pleasant than paintings with simple IM (69% vs 77%, *X*^*2*^(1) = 7.26, *p* < 0.01) and no-IM (69% vs 75%, *X*^*2*^(1) = 4.37, *p* < 0.05), maybe because motion perception is induced in an excessively unrealistic way. No differences between percentages of pleasantness for human vs non-human representations are found in each motion category (all *X*^*2*^ tests yielded to *p* > 0.05). Figurative paintings are judged as more pleasant than abstract ones, both for static and dynamic categories (No-IM: 75% vs 54%, *X*^*2*^(1) = 32.41, *p* < 0.00001; IM: 77% vs 65%, *X*^*2*^(1) = 10.51, *p* < 0.01), confirming that non-expert observers (like ours) prefer figurative art. Abstract dynamic paintings are rated more pleasant than static ones (65% vs 54%, *X*^*2*^(1) = 5.86, *p* < 0.05), maybe due to positive aesthetic appreciation of dynamism in artworks. All paintings, regardless of their style, are also rated more pleasant than optical illusions, which are pleasant only for 52% of the observers (all *X*^*2*^-tests yielded to *p* < 0.01). Although the amount of pleasantness varies across categories, it does not seem to influence pupil responses. A two-way ANOVA–type of motion and pleasantness—shows no effect of aesthetic pleasantness, neither for figurative (F(1,20) = 0.51, *p* > 0.05, η^2^ = 0.001) or abstract paintings (F(1,19) = 0.70, *p* > 0.05, η^2^ = 0.005). Only the type of motion has a significant effect on the pupil response (F(2,40) = 4.54, *p* < 0.01, η^2^ = 0.13 for figurative stimuli, F(1,19) = 9.05, *p* < 0.01, η^2^ = 0.20 for abstract stimuli), showing that what changes the pupil diameter is the motion interpretation in the scene and not the appreciation of the painting. There are no differences in pupil size between participants who liked the illusions and those who did not (*t*(18) = -0.18, *p* > 0.05).

In Fig 5B, mean pupil responses have been replotted, for each category, according to the subjective familiarity for the paintings. Independently on motion categories, on average, it appears to be no difference in familiarity between figurative and abstract artworks (18% vs 20%, *X*^*2*^(1) = 0.31, *p* > 0.05), whereas optical illusions are much more known compared to figurative (43% vs 18%, *X*^*2*^(1) = 70.18, *p* < 0.0001) and abstract paintings (43% vs 20%, *X*^*2*^(1) = 37.80, *p* < 0.0001), probably because they are widespread on the internet and books. Paintings with *enhanced* IM are the less known (11%) with respect to the other figurative and abstract paintings (all *X*^*2*^-tests yielded to *p* < 0.05). There is no difference in familiarity between static and dynamic abstract paintings (23% vs 18%, *X*^*2*^(1) = 0.14, *p* > 0.5). Dynamic IM figurative paintings are more familiar than dynamic abstract ones (25% vs 18%, *X*^*2*^(1) = 4.24, *p* < 0.5). No differences between percentages of familiarity for human vs non-human figurative paintings are found in each motion category (all *X*^*2*^ tests have *p* values > 0.05). These data confirm that our participants can be considered non-expert in art since they do not know most of the artworks (on average 81% of the paintings are unfamiliar). No influences of familiarity on pupil responses are found. A two-way ANOVA–type of motion and familiarity—does not show any significant difference in pupil size between participants who recognized the paintings as familiar and those who did not, neither for figurative (F(1,17) = 1.48, *p* > 0.05, η^2^ = 0.007) and abstract paintings (F(1,14) = 1.006, *p* > 0.05, η^2^ = 0.02). Only the type of motion has a significant effect on the pupil response (F(2,34) = 3.74, *p* < 0.05, η^2^ = 0.08 for figurative stimuli, F(1,14) = 10.19, *p* < 0.01, η^2^ = 0.07 for abstract stimuli), showing that what changes the pupil diameter is the motion interpretation in the scene and not the familiarity for the painting. There are no differences in pupil sizes between participants who were familiar with the illusions and those who did not (*t*(16) = 0.53, *p* > 0.05).

## Discussion

In the present study we recorded pupillary responses to implied motion depicted in figurative and abstract paintings. Figurative paintings showing static subjects and abstract artworks conveying the perception of stillness have been presented as controls for implied motion. Pupil responses to movies (figurative and abstract) and motion illusions have been also measured in order to compare the effect of depicted implied motion with that of real and illusory motion. The average luminance of all stimuli was the same and lower than that of the background, allowing us to measure the effect of the different types of motion independently of PLR.

Our results show that all figurative stimuli (movies and paintings), showing human and non-human subjects, induce an increase in pupil size with a different amount of dilation based on motion strength. Perception of figurative real motion induces the greatest pupillary dilation compared with static stimuli with and without implied motion, confirming previous findings [38]. Perception of motion in figurative paintings induces a dilation that increases with its strength: *enhanced* implied motion (blur, motion streaks, speed lines, etc.) induces a larger pupil size than simple implied motion (action freezing), similar to that induced by real motion. Both types of dynamic paintings induce more dilation than paintings of static subjects, although only the difference with *enhanced* motion paintings is significant, suggesting again that the more evident the motion in the stimulus, the larger the pupil dilation. This same effect is also found in those observers that misinterpreted the motion in a given figurative painting: pupil size is larger for those observers that consider it as dynamic with respect to those that consider it as static.

As already suggested [38], pupillary responses to real motion could be attributed to the activation of the sympathetic system [78]. That is, the observed dilation could reflect a physiological response of the system preparing for a dynamic situation causing arousal [8–11,79]. We can also speculate that the pupil increases its diameter in order to widen the visual field [80] to better perceive the objects’ movement in the periphery [81]. The same hypotheses might explain pupil responses to figurative implied motion, which simulate the movement of realistic subjects in the scene. The different amount of pupil dilation to real and implied motion of figurative subjects may reflect different degrees of activation of the cortical area MT, dedicated to the processing of both real motion [82–87] and implied motion information in photos [88–92] and paintings [49,93–95]. Also, the difference between *enhanced* and simple implied motion in paintings may depend on the degree of MT activation, since it increases with the strength of implied motion in images [96]. The hypothesis that pupil dilation for figurative implied motion actually depends on the processing of the embodied motion information in the paintings is also supported by the fact that, for a given artwork, pupil size is larger for those observers that consider it as dynamic with respect to those that consider it as static. Thus, pupil responses depend on the subjective interpretation of dynamism in complex images, as seen previously [38], confirming previous studies finding that the subjective interpretation of complex images has a relevant effect on pupil diameter [29,31,37,38,68,97].

Since our paintings have the same mean luminance as the photos used in previous studies [38], it is possible to compare pupil responses to stimuli showing the same types of figurative subjects but differing for the realism of their representations. The effect found here with figurative paintings is comparable to that obtained with realistic photographs, although the effect sizes, estimated by Cohen’s d statistics, shows that the magnitude of the differences between static and dynamic stimuli is larger in photos than in paintings [38]. This is probably due to the fact that, albeit photos and figurative paintings show realistic static or dynamic subjects, paintings are not faithful reproductions of reality, but rather artistic representations mediated by the artist mind and its technique. We conclude that figurative implied motion in paintings induce dilation as real movies, although to a lower extent, and this effect is similar to that observed with photographs [38]. Overall, these results suggest that motion processing influences the pupil diameter even if the motion information is embodied in very complex and ambiguous stimuli as artworks. Differently from the previous study [38], here we do not find differences in pupil responses to paintings showing human versus non-human subjects.

Motion illusions induce pupil constriction with respect to real and implied motion, as shown before [38]. The pupil size of observers that rated the illusion as dynamic is also smaller than the pupil of those that consider it as static, confirming that the observed constriction actually depends on the subjective perception of apparent motion.

The pupillary constriction for illusory motion does not seem to be due to the specific features of illusions, since images sharing the same colors and shapes with illusions without conveying the impression of motion, have been shown to induce dilation as expected from their physical luminance [38]. The different effects induced by illusory motion compared to that produced by real and figurative implied motion cannot be explained either by differences in the degree of activation of MT [98–100], or by time differences of MT+ response [100]. It could be that the extraction of motion information from artificial static stimuli with no realistic subjects in action requires a higher attention load. It has been found that non-visual cortical areas, that are part of an extended attentional network, are activated during observation of motion illusions, but not during the presentation of real movies [99]. Involvement of multiple areas could be explained by considering that the perception of illusory motion, arises through the cognitive combination of several image features [101,102]. Moreover, the perception of real motion has a stronger adaptive valence for survival than the sensation of motion in artificial stimuli, leading to fine-tuned dedicated brain mechanisms [84,85,87,103–108], Thus, it could be that illusory motion, relying on the relative contribution of several parts of the brain [99], induces different pupil responses compared to real figurative and implied motion elaborated by specific motion areas.

The trend of pupil responses for abstract stimuli is also different from figurative stimuli. Abstract real motion induces pupil dilation, larger than that induced by all the other abstract categories, as observed for figurative movies. However, abstract paintings depicting dynamism induces less dilation than paintings conveying the perception of stillness. This effect is the exact opposite and of a larger magnitude than that obtained with figurative paintings or photos [38], where dynamic realistic subjects induce more dilation than static subjects. Thus, it seems that the processing of abstract motion induces pupil constriction compared to processing of stillness. We found the same effect by analysing the results of miscategorizations: for a given abstract artwork, pupil size is smaller for those observers that consider it as dynamic with respect to those that consider it as static, confirming again the influence of subjective interpretation on pupil modulation. One could note that the effect of abstract dynamic paintings is similar to that produced by illusory motion, although less powerful.

The explanation of the different effect on the pupil of abstract dynamic paintings with respect to all other painting categories (figurative static and dynamic and even abstract static) pose a challenge since there are no other studies investigating pupil responses to non-figurative stimuli. A possible explanation could be these paintings may be considered more complex and difficult to be understood than all others. However, this explanation is not consistent with the existing literature showing that a large cognitive effort induces pupil dilation [15,109]. We could argue that in abstract paintings, as in illusions, the impression of motion is conveyed by structural features such as curved lines, shapes, orientations, colors contrast, asymmetry, etc. For this reason, the explanations proposed above for the constriction effect of illusory motion with respect to that of real motion and figurative implied motion might be held for abstract implied motion as well. That is the processing of figurative motion may have a more adaptive valence and a consequent specialization of dedicated cortical areas compared to abstract motion, which may be elaborated by a more extended cortical network. In fact, the effect could be dependent on different top-down cortical modulations involving MT, that is activated by both abstract and representational dynamic paintings [49,93], and other non-visual areas. Indeed, in figurative paintings, the observers may interpret the motion sequence of an object, infer the intentions of the subjects, imaging the narration of the event, and so on. This suggests that, when the perception of dynamism derives from the intrinsic realistic characteristics of the depicted subject, many areas in charge of objects’ recognition, attention, imagination, memory, familiarity, etc., are also involved, possibly leading to the increase of pupil size. Instead, when motion impression is conveyed only by image structural features (lines and shapes orientations, colors combinations, particular compositional characteristics such as balance), other areas, involved in processing of low-level features (colors, contrast, symmetry, etc.), may be activated, leading to a decrease of pupil size. This is supported by studies suggesting that in abstract paintings, compared to the representational ones, attention is more focused on low-level visual features [110,111].

Although the mean luminance was strictly matched across stimulus categories, its spatial distribution could be variable and in principle affects pupil size differences [112]. However, from a visual inspection of our stimuli (S1 List), it appears that there are very slight differences between categories in the positions of bright areas that can be located either in the center of the stimulus, corresponding to the fovea, or more peripherally. Anyway, past works have shown that pupil responses did not depend on the position of very bright light sources [36,37]. Finally, a possible effect of the luminance distribution on our result is unlikely, given the small size of our images (~9°x7°) and the large, bilateral receptive field sizes of the brainstem neurons driving pupillary constrictions in response to luminance increments [113].

Our data also exclude the possibility that pupil responses for the different types of motion stimuli are influenced by observers’ eye movement.

The subjective evaluation of motion in our stimuli reveals that it is easier to perceive motion in figurative than in abstract paintings. This suggests that abstract artworks are more ambiguous, and that the extraction of motion information is easier when real-life subjects are represented (a moving car, a running man, etc.). Illusions, on the other hand, even though structurally similar to abstract paintings, give way to impression of motion most of the times: just a small percentage of participants did not perceive motion in some of the illusions, as reported previously [114]. Interestingly the number of cases in which our participants miscategorized figurative paintings (5.3%) is compatible with that found with realistic photos [38], suggesting that observers are very good at extracting motion information even from very complex and ambiguous images as artworks. The higher percentage of miscategorizations found for photos of humans compared to objects or animals [38], revealing our tendency to rate humans as more dynamic, does not emerge here with paintings.

Appreciation and pleasantness, being associated with a general state of arousal [115,116], produce the activation of the sympathetic system and therefore induce pupil dilation [61]. However, here, they do not seem to be responsible for the observed modulation of the pupil. For example, abstract dynamic paintings induce constriction despite being considered more pleasant than static.

In a similar way, familiarity, that has been shown to increase pupil size [62,63], cannot explain the different effects produced by our stimuli. Therefore, if familiarity was responsible for the effects on the pupil, we should expect more dilation for illusions, that, instead, induced the smallest pupil diameter of all other stimuli.

## Conclusions

Overall, our results show that real motion perception induces pupillary dilation independently on the type of moving subjects, figurative or abstract. Even motion information embodied in very complex stimuli as artworks can influence pupil size, and even when paintings have no real-life motion cues, as in abstract paintings. Interestingly, the effect of implied motion depends on the way motion perception is conveyed: pupil size increases by showing realistic figures in action (humans, objects, animals), whereas pupil size decreases by using particular image features to create the impression of motion (colors, asymmetry, contrast, balance, etc.). This suggests the involvement high-level visual processing recruiting distinct mechanisms in the analysis of figurative and abstract implied motion. All these effects depend on the subjective interpretation of the stimuli, providing further evidence that pupil size is not merely regulated by the subcortical system responsible for the PLR, but it is also influenced by top-down cortical modulations.

## Supporting information

S1 List List of paintings used as stimuli. Title, artist, year and web source are reported for each painting. Paintings are divided by type of content (abstract, non-human, humans) and motion category (No-IM, IM, *Enhanced*-IM). Some images have been cut with respect to the originals to better fit with the assigned nominal category.
(XLSX)
